# Serum Concentrations of Citrate, Tyrosine, 2- and 3- Hydroxybutyrate are Associated with Increased 3-Month Mortality in Acute Heart Failure Patients

**DOI:** 10.1038/s41598-019-42937-w

**Published:** 2019-05-01

**Authors:** Sarah Stryeck, Michaela Gastrager, Vesna Degoricija, Matias Trbušić, Ines Potočnjak, Bojana Radulović, Gudrun Pregartner, Andrea Berghold, Tobias Madl, Saša Frank

**Affiliations:** 1 Gottfried Schatz Research Center for Cell Signaling, Metabolism and Aging, Molecular Biology and Biochemistry, Medical University of Graz, Neue Stiftingtalstraße 6/6, 8010 Graz, Austria; 2 University of Zagreb School of Medicine, Šalata 3, 10000 Zagreb, Croatia; 3 University Hospital Centre Sisters of Charity, Department of Medicine, Vinogradska 29, 10000 Zagreb, Croatia; 4 University Hospital Centre Zagreb, Kišpatićeva 12, 10000 Zagreb, Croatia; 5 Institute for Medical Informatics, Statistics und Documentation, Medical University of Graz, Auenbruggerplatz 2, 8036 Graz, Austria; 6 BioTechMed-Graz, Mozartgasse 12/II, 8010 Graz, Austria

**Keywords:** Prognostic markers, Heart failure, Outcomes research

## Abstract

Considering the already established relationship between the extent of the metabolic dysfunction and the severity of heart failure (HF), it is conceivable that the metabolomic profile of the serum may have a prognostic capacity for 3-month mortality in acute heart failure (AHF). Out of 152 recruited patients, 130 serum samples were subjected to the metabolomic analyses. The 3-month mortality rate was 24.6% (32 patients). Metabolomic profiling by nuclear magnetic resonance spectroscopy found that the serum levels of 2-hydroxybutyrate (2-HB), 3-hydoxybutyrate (3-HB), lactate, citrate, and tyrosine, were higher in patients who died within 3 months compared to those who were alive 3 months after onset of AHF, which was confirmed by univariable logistic regression analyses (p = 0.009, p = 0.005, p = 0.008, p<0.001, and p<0.001, respectively). These associations still remained significant for all tested metabolites except for lactate after adjusting for established prognostic parameters in HF. In conclusion, serum levels of 2-HB, 3-HB, tyrosine, and citrate measured at admission are associated with an increased 3-month mortality rate in AHF patients and might thus be of prognostic value in AHF.

## Introduction

Heart failure (HF) is a final stage of various cardiovascular diseases and a common cause of disability and death^1^. The European Society of Cardiology (ESC) defines HF as an abnormality of the cardiac structure and function, which results in a diminished oxygen supply to the metabolizing tissues^2,3^. Acute heart failure (AHF) is primarily characterized by the rapid onset of symptoms and signs of HF^3^.

Metabolic dysfunction, an inherent feature of the HF pathophysiology^4,5^, reflects not only the altered metabolism of the myocardium but rather overall contributions from peripheral tissues and organs^6^. Hemodynamic impairment and the thereby accompanied tissue hypoperfusion and congestion increase the serum levels of catecholamines^7^, inflammatory cytokines^8^, and natriuretic peptides^9^. These promote lipolysis, proteolysis, and oxidative stress, the hallmarks of metabolic dysfunction in HF^5,10^ either directly or via the induction of insulin resistance, a principal metabolic feature of the HF pathophysiology^11^. Metabolic dysfunction and the catabolic dominance in HF are further intensified by a reduced appetite and an impaired intestinal nutrient absorption due to congestion and intestinal edema as well as the diminished biosynthetic capacity of the hypo-perfused and/or congested liver^12–15^.

The extent of the metabolic perturbation parallels the hemodynamic impairment, i.e. the severity of HF. Accordingly, it is conceivable that the metabolomic profile of the patients’ serum at admission may have a valuable prognostic potential for 3-month mortality in AHF. Untargeted metabolic profiling using ^1^H nuclear magnetic resonance (NMR) spectroscopy has been successfully established in the recent years for a wide range of metabolites and biological matrices, including human biofluids^16–20^. The aim of the present study was therefore to employ NMR spectroscopy to identify serum metabolites that are prognostic for 3-month mortality in patients with AHF.

## Results

### Clinical characteristics, pre-admission medication and comorbidities

Out of 152 AHF patients recruited in the study^21–24^, serum samples of 130 patients were subjected to the metabolomic analyses. Of these, 32 (24.6%) died within three months after onset of AHF. As presented in Table 1, body mass index (BMI) and weight were significantly lower and the liver enlargement and peripheral edema, were considerably more frequently observed in patients who did not survive compared to those who survived the first three months after onset of AHF. These groups were not significantly different regarding age, gender, smoking, New York Heart Association Functional Classification (NYHA), mean arterial pressure (MAP), heart rate, ejection fraction, or systolic pulmonary artery pressure. The incidence of distended jugular veins and ascites as well as pre-admission medication were also similar in both groups (Table 1). Additionally, the groups did not differ with respect to the history of hyperlipidemia, hypercholesterolemia, hypertension, type 2 diabetes mellitus (T2DM), chronic obstructive pulmonary disease, chronic kidney disease, cardiomyopathy, or acute coronary syndrome (Supplementary Table 1).

**Table 1:** Baseline characteristics and pre-admission medication of AHF patients according to survival status after three months.

	All (N = 130)	Alive (N = 98)	Dead (N = 32)	p-value
Age (years)	77.1 (45.5–96.7)	75.2 (45.5–93.0)	78.8 (50.9–96.7)	0.263
Female	67 (51.5%)	47 (48.0%)	20 (62.5%)	0.162
BMI (kg/m^2^)	28.4 (17.1–43.5)	29.0 (19.9–42.6)	25.4 (17.1–43.5)	**0.017**
Weight (kg)	80.0 (40.0–144.0)	84.5 (46.0–135.0)	72.5 (40.0–144.0)	**0.001**
Smoking	34 (26.2%)	28 (28.6%)	6 (18.8%)	0.356
NYHA class				
2	11 (8.5%)	10 (10.2%)	1 (3.1%)	0.454
3	73 (56.2%)	55 (56.1%)	18 (56.2%)	
4	46 (35.4%)	33 (33.7%)	13 (40.6%)	
MAP (mmHg)	103.3 (65.0–160.0)	103.3 (65.0–160.0)	96.7 (70.0–150.0)	0.051
Heart rate (beats/min)	100.0 (36.0–160.0)	102.0 (36.0–160.0)	93.0 (60.0–140.0)	0.087
EF (%)	45.0 (20.0–70.0)	45.0 (20.0–70.0)	40.0 (20.0–70.0)	0.412
SPAP (mmHg)	45.0 (35.0–80.0)	45.0 (35.0–80.0)	50.0 (35.0–70.0)	0.144
JVD	46 (35.4%)	31 (31.6%)	15 (46.9%)	0.138
Enlarged liver	47 (36.2%)	30 (30.6%)	17 (53.1%)	**0.033**
Peripheral edema	89 (68.5%)	62 (63.3%)	27 (84.4%)	**0.029**
Ascites	20 (15.4%)	16 (16.3%)	4 (12.5%)	0.780
Statins	34 (26.2%)	24 (24.5%)	10 (31.2%)	0.490
ß-blockers	59 (46.5%)	41 (42.7%)	18 (58.1%)	0.152
ACEI	74 (56.9%)	56 (57.1%)	18 (56.2%)	1.000
Amplodipine	22 (17.1%)	19 (19.4%)	3 (9.7%)	0.279

Data are presented as n (%) or as median and range (minimum to maximum). Differences between the two groups were tested with Fisher’s exact test or the Mann-Whitney U test; significant differences are depicted in bold.

AHF, acute heart failure; BMI, body mass index; EF, ejection fraction; JVD, jugular venous distension; MAP, mean arterial pressure; NYHA, New York Heart Association Functional Classification SPAP, systolic pulmonary artery pressure.

### Laboratory parameters

While glomerular filtration rate (GFR) and total cholesterol were significantly lower, the serum levels of urea, creatinine, N-terminal pro brain natriuretic peptide (NT-proBNP), alanine aminotransferase (ALT), and aspartate aminotransferase (AST) were significantly higher in AHF patients who died within three months compared to those who survived three months after onset of AHF (Table 2). The two groups did not differ in the serum levels of proteins, albumin, interleukin-6 (IL-6), C-reactive protein (CRP), as well as low-density lipoprotein cholesterol (LDL cholesterol), high-density lipoprotein cholesterol (HDL-cholesterol), and triglycerides (Table 2).

**Table 2:** Laboratory parameters of AHF patients according to survival status after three months.

	All (N = 130)	Alive (N = 98)	Dead (N = 32)	p-value
GFR (ml/min/1.73 m^2^)	51.3 (15.0–105.7)	55.1 (16.0–105.7)	42.3 (15.0–77.0)	**0.002**
Urea (mmol/L)	8.0 (3.0–64.0)	8.0 (3.0–64.0)	14.0 (4.0–41.0)	**<0.001**
Creatinine (µmol/L)	106.0 (53.0–273.0)	102.5 (53.0–255.0)	126.0 (66.0–273.0)	**0.024**
NT-proBNP (ng/mL)	8220.5 (171–70000)	6304 (171–70000)	15683.5 (3903–46054)	**<0.001**
ALT (U/L)	23.0 (6.0–623.0)	20.0 (6.0–623.0)	25.0 (13.0–556.0)	**0.008**
AST (U/L)	27.0 (10.0–666.0)	25.0 (10.0–666.0)	36.5 (15.0–487.0)	**0.001**
Serum protein (g/L)	68.0 (31.0–87.0)	68.0 (31.0–87.0)	65.0 (53.0–79.0)	0.076
Albumin (g/L)	40.0 (22.0–62.0)	40.0 (22.0–62.0)	36.0 (24.0–62.0)	0.085
IL-6 (pg/mL)	18.9 (0.4–300.0)	18.4 (0.4–300.0)	24.4 (1.2–300.0)	0.283
CRP (µg/mL)	9.0 (0.2–247.4)	7.7 (0.2–247.4)	13.0 (1.1–169.0)	0.169
Total cholesterol (mmol/L)	3.8 (1.7–9.1)	4.2 (1.7–9.1)	3.6 (2.1–6.9)	**0.018**
LDL cholesterol (mmol/L)	2.3 (0.8–6.3)	2.4 (1.0–6.3)	2.0 (0.8–4.7)	0.067
HDL cholesterol (mmol/L)	1.0 (0.3–3.6)	1.0 (0.4–3.6)	0.8 (0.3–2.3)	0.053
Triglycerides (mmol/L)	1.1 (0.6–4.3)	1.1 (0.6–4.3)	1.0 (0.6–3.0)	0.179

Data are presented as median and range (minimum to maximum). Differences between AHF patients who died and those who survived the first three months after onset of AHF were tested with the Mann-Whitney U test; significant differences are depicted in bold.

ALT, alanine aminotransferase; AST, aspartate aminotransferase; AHF, acute heart failure; CRP, C-reactive protein; GFR, glomerular filtration rate; IL-6, interleukin-6; LDL, low-density lipoprotein; HDL, high-density lipoprotein; NT-proBNP, N-terminal pro brain natriuretic peptide.

### Identification and prognostic value of metabolites differently abundant in patients who were alive and those who died within three months after onset of AHF

**Figure 1:**
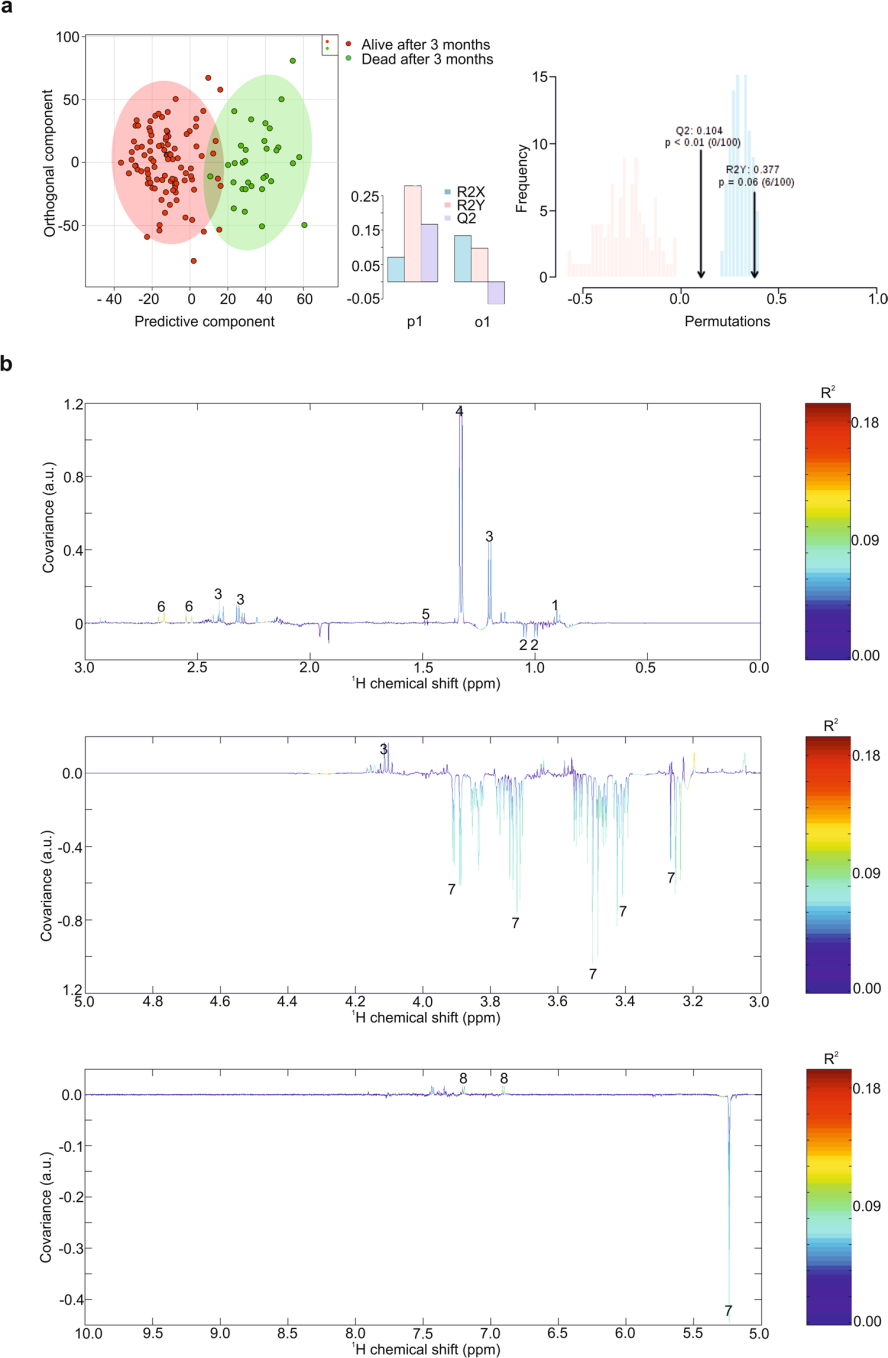
OPLS-DA plot of AHF serum samples. (**a**) Multivariate OPLS-DA plot of 3-month mortality. (**b**) Reduced NMR spectra reveal altered metabolites in normalized AHF serum samples. Positive covariance corresponds to metabolites present at increased concentrations, whereas negative covariance corresponds to decreased metabolite concentrations in patients that died within three months. Predictivity of the model is represented by R^2^. 1…2-hydroxybutyrate, 2…valine, 3…3-hydroxybutyrate, 4…lactate, 5…alanine, 6…citrate, 7…glucose, 8…tyrosine.

In order to assess metabolic differences between patients that did and did not die within three months after onset of AHF, nuclear magnetic resonance (NMR) metabolic profiling of 130 serum samples was performed. When comparing differences in metabolic fingerprints between patients who were alive and those who died within 3 months after onset of AHF, Orthogonal-Partial Least Squares - Discriminant Analysis (O-PLS-DA) revealed a slight clustering of patient samples with correlation coefficients R^2^ up to 0.18 and a Q^2^ of 0.104 (p < 0.01) (Fig. 1a). Reduced NMR spectra revealed altered metabolites in normalized AHF serum samples (Fig. 1b) and indicated that the levels of 2-hydroxybutyrate (2-HB), 3-hydroxybutyrate (3-HB), lactate, alanine, citrate, and tyrosine were higher, whereas valine and glucose were lower in patients who died within three months after onset of AHF.

**Figure 2:**
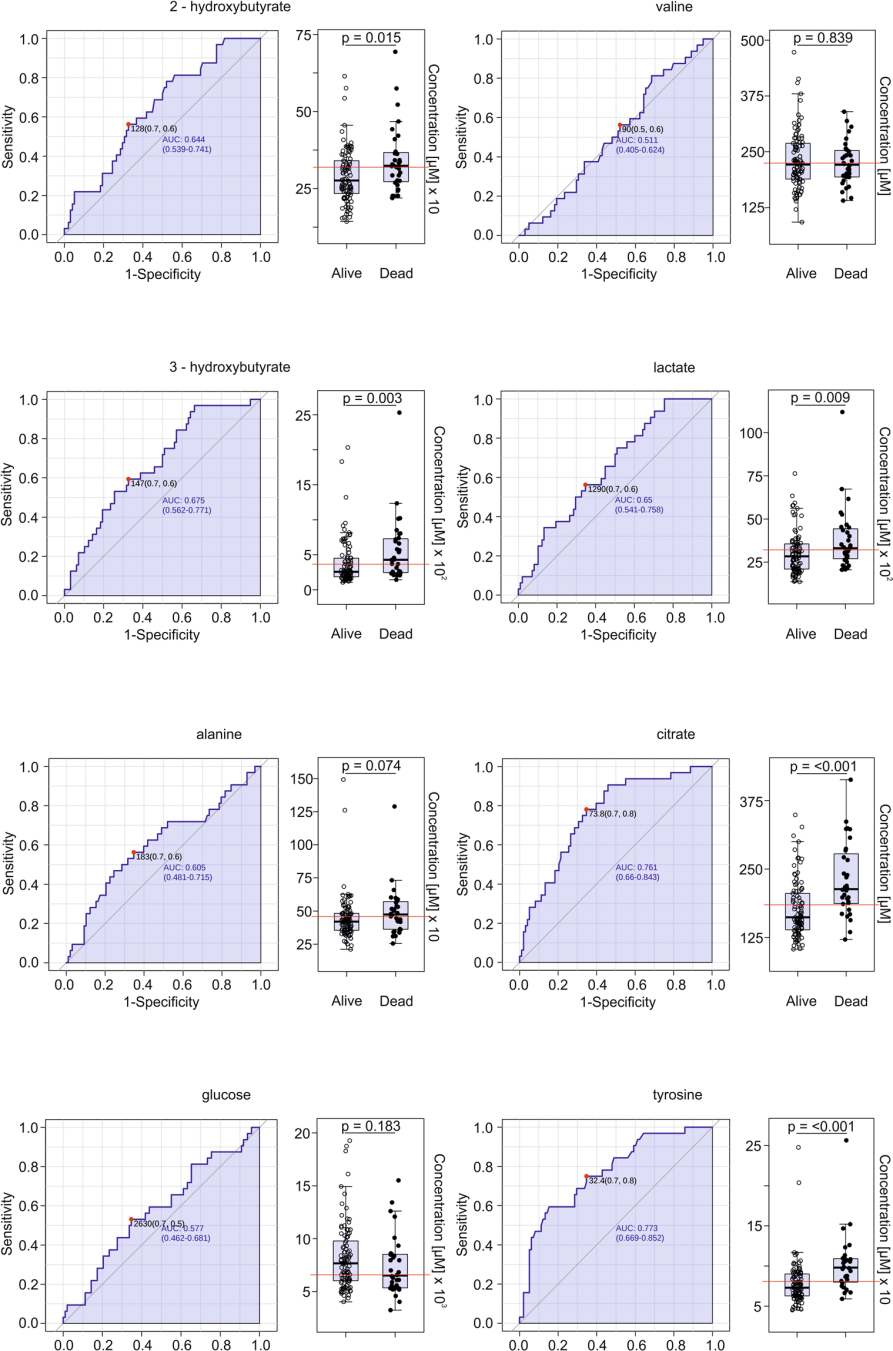
Serum levels and ROC analysis of altered metabolites. Differences between patients who were alive and those who died within three months after onset of AHF were tested with the Mann-Whitney U test. Absolute concentrations were used to calculate ROC curves for distinct metabolites and to assess the prognostic value of the distinct metabolites for 3-month mortality.

In order to assess the prognostic value of these metabolites, the absolute concentrations were determined. As shown in Fig. 2 the levels of 2-HB, 3-HB, lactate, tyrosine, and citrate, but not of alanine, valine, and glucose, were significantly different (increased) in patients who died compared to those who survived the first three months after onset of AHF. Furthermore, sensitivity and specificity for each metabolite were assessed using Receiver Operating Characteristic (ROC) curves (Fig. 2). The highest values for the area under the curve (AUC), indicating highest predictivity, were obtained for tyrosine and citrate, followed by 3-HB, lactate, 2-HB, alanine, glucose, and valine (Fig. 2).

### Correlation of 2-HB, 3-HB, lactate, citrate, and tyrosine concentrations with laboratory and clinical parameters

To examine the relationship between the identified metabolites, whose serum levels were significantly higher in patients who did not survive compared to those who survived the first three months after onset of AHF, and clinical and laboratory parameters, correlation analyses were performed. As shown in Table 3, the serum concentration of citrate, but not of any other metabolite, was significantly positively correlated with age, urea and creatinine as well as negatively correlated with fibrinogen, and HDL cholesterol. Both citrate and tyrosine were significantly negatively correlated with BMI, MAP, total cholesterol, LDL cholesterol, and triglyceride levels. Citrate and 3-HB were negatively correlated with GFR but positively with urea. Additionally, 2-HB, 3-HB, citrate, and tyrosine were significantly positively correlated with NT-proBNP. While only 2-HB and lactate were significantly positively correlated with ALT, all tested metabolites were significantly positively correlated with AST. Serum concentrations of 3-HB, citrate, and tyrosine, but not of 2-HB and lactate, were furthermore significantly positively correlated with concentrations of IL-6.

**Table 3:** Correlation analyses of 2-HB, 3-HB, lactate, citrate and tyrosine with clinical and laboratory parameters.

Metabolites (µmol/L)							
		2-HB	3-HB	Lactate	Citrate	Tyrosine	n
Age (years)	r	−0.04	0.07	−0.04	0.27	0.06	130
	p	0.627	0.430	0.690	**0.002**	0.513	
BMI (kg/m^2^)	r	0.00	−0.12	−0.12	−0.22	−0.23	130
	p	0.989	0.185	0.162	**0.012**	**0.009**	
MAP (mm Hg)	r	−0.12	−0.07	−0.04	−0.27	−0.28	130
	p	0.163	0.398	0.665	**0.002**	**0.001**	
NT-proBNP (pg/mL)	r	0.18	0.25	0.13	0.43	0.28	124
	p	**0.043**	**0.004**	0.141	**<0.001**	**0.002**	
GFR (ml/min/1.73 m^2^)	r	−0.10	−0.17	−0.15	−0.57	−0.12	129
	p	0.243	**0.049**	0.085	**<0.001**	0.183	
Urea (mmol/L)	r	0.14	0.22	−0.01	0.48	0.10	129
	p	0.111	**0.013**	0.901	**<0.001**	0.274	
Creatinine (mol/L)	r	0.13	0.17	0.13	0.51	0.09	129
	p	0.139	0.050	0.147	**<0.001**	0.333	
ALT (U/L)	r	0.19	0.16	0.19	0.03	0.16	128
	p	**0.034**	0.064	**0.032**	0.746	0.080	
AST (U/L)	r	0.21	0.27	0.29	0.20	0.32	129
	p	**0.019**	**0.002**	**0.001**	**0.025**	**<0.001**	
Fibrinogen (g/L)	r	0.13	−0.07	−0.09	−0.38	−0.13	126
	p	0.149	0.447	0.311	**<0.001**	0.148	
IL-6 (pg/mL)	r	0.09	0.18	0.12	0.22	0.22	130
	p	0.285	**0.045**	0.188	**0.010**	**0.013**	
Total cholesterol (mmol/L)	r	−0.02	−0.17	0.03	−0.44	−0.21	130
	p	0.802	0.054	0.726	**<0.001**	**0.018**	
LDL-cholesterol (mmol/L)	r	−0.01	−0.14	0.05	−0.35	−0.19	130
	p	0.898	0.125	0.545	**<0.001**	**0.030**	
HDL-cholesterol (mmol/L)	r	−0.04	−0.03	0.10	−0.23	−0.06	130
	p	0.686	0.745	0.274	**0.009**	0.470	
Triglycerides (mmol/L)	r	0.02	−0.12	0.01	−0.23	−0.19	130
	p	0.829	0.187	0.929	**0.009**	**0.029**	

Data are presented as Spearman correlation coefficient r, p-value, and number of available samples (n); significant correlations are depicted in bold.

ALT, alanine aminotransferase; AST, aspartate aminotransferase; BMI, body mass index; GFR, glomerular filtration rate; HDL, high-density lipoprotein; 2-HB, 2-Hydroxybutyrate; 3-HB, 3-Hydroxybutyrate; IL-6, interleukin 6; LDL, low-density lipoprotein; MAP, mean arterial pressure; NT-proBNP, N-terminal pro brain natriuretic peptide.

### Logistic regression analyses

To further examine the association of the metabolites with 3-month mortality, we performed logistic regression analyses (Table 4) for 2-HB, 3-HB, lactate, tyrosine, and citrate, the metabolites that were significantly higher (Fig. 2) in patients who did not survive three months after onset of AHF as compared to those that did. As shown in Table 4, the univariable analyses showed a significant positive association of the serum levels of all tested metabolites with 3-month mortality. These associations remained significant for all tested metabolites except lactate, upon adjusting for the established prognostic parameters in HF, namely age, sex, BMI, T2DM, NT-proBNP, GFR, MAP, and LDL cholesterol.

**Table 4:** Logistic regression analyses of 3-month mortality for 2-HB, 3-HB, lactate, citrate, and tyrosine.

	Unadjusted		Adjusted^a^	
	OR (95% CI)	p-Value	OR (95% CI)	p-Value
2-HB (µmol/L)	7.14 (1.73–33.50)	**0.009**	10.29 (1.78–74.18)	**0.013**
3-HB (µmol/L)	2.42 (1.32–4.58)	**0.005**	2.15 (1.06–4.50)	**0.034**
Lactate (µmol/L)	4.36 (1.52–13.57)	**0.008**	3.64 (1.01–14.92)	0.057
Citrate (µmol/L)	26.24 (5.89–139.62)	**<0.001**	11.74 (1.44–113.20)	**0.026**
Tyrosine (µmol/L)	24.48 (5.11–157.78)	**<0.001**	34.70 (4.49–386.70)	**0.002**

^a^The model was adjusted for age, sex, BMI, T2DM, NT-proBNP, GFR, MAP and LDL-cholesterol.

Log-transformed values of the metabolite concentrations were used as covariates.

The unadjusted analyses comprised data of 130 patients (32 events) and the adjusted analyses data of 122 patients (29 events).

Significant results are depicted in bold.

BMI, body mass index; 2-HB, 2-Hydroxybutyrate; 3-HB, 3-Hydroxybutyrate; CI, confidence interval; LDL, low-density lipoprotein; MAP, mean arterial pressure; NT-proBNP, N-terminal pro brain natriuretic peptide; OR, odds ratio.

## Discussion

Despite established multivariable predictive models comprising patients’ characteristics, clinical signs and serum biomarkers, the estimation of risk in AHF is difficult, not accurate and poorly applicable in daily clinical practice^25^. Therefore, the identification of new biomarkers, which are related to the complex mechanisms of the AHF pathophysiology, may help in identifying high risk patients and initiating timely therapeutic interventions.

Considering a positive relationship between metabolic dysfunction, which is an established inherent feature of HF, and the extent of hemodynamic impairment, serum metabolites might be useful prognostic markers in AHF^5,26,27^.

In the present study, we show, that serum levels of 2-HB, 3-HB, citrate, and tyrosine are independently associated with 3-months mortality in AHF patients, even after adjusting for other well-known risk factors. Increased levels of these metabolites as well as lactate in AHF patients who did not survive the first three months after onset of AHF strongly indicate a more severe state of the disease and more severe systemic metabolic perturbations in this group of AHF patients, compared to AHF patients who survived 3 months after onset of AHF. Indeed, with the exception of lactate the levels of 2-HB, 3-HB, citrate, and tyrosine were significantly positively correlated with NT-proBNP, reflecting a positive relationship with the severity of HF^28^.

It is well established that a decreased cardiac output and a subsequent decreased tissue perfusion, a consequence of left-sided HF, congestion, are accompanied by increased serum levels of catecholamines, natriuretic peptides and inflammatory cytokines. These, in turn trigger lipolysis, proteolysis, and oxidative stress, either directly or via the induction of insulin resistance^7–11^. Increased 2-HB levels have been shown to reflect insulin resistance and tricarboxylic acid (TCA) cycle overload^29–31^ as well as increased oxidative stress^32,33^. We observed increased serum levels of 2-HB in patients who died within three months after onset of AHF, which argues for the role of insulin resistance and oxidative stress in metabolic perturbations and mortality in our AHF cohort. This is further substantiated by increased 3-HB levels in these patients, since its hepatic synthesis and serum levels are known to be augmented in states of insulin resistance and excess fatty acid supply^31^. Alternatively or additionally, increased 3-HB serum levels in this group of AHF patients may be a consequence of the decreased utilization of 3-HB as an energy substrate in skeletal muscle. Previous studies have shown decreased 3-HB utilization in skeletal muscle of HF patients compared to healthy controls^34^.

Elevated serum 3-HB levels may result in an increased uptake and oxidation by the failing heart. This in turn may diminish the uptake and utilization of glucose^35^, whose energy producing efficiency outperforms that of 3-HB^36^. Accordingly, by displacing glucose utilization, the elevated 3-HB might be detrimental when oxygen provision is limited due to decreased cardiac tissue perfusion and congestion, as is encountered in the failing heart. Furthermore, an increased intracellular pool of acetyl CoA, secondary to an increased 3-HB oxidation, may facilitate a hyperacetylated state in cardiac myocytes^37^. This in turn may lead to a posttranslational modification of enzymes involved in cellular energy metabolism, causing detrimental metabolic perturbations in the failing heart.

However, 3-HB has been shown to promote the myocardial blood flow in healthy humans^35^, and 3-HB is a natural inhibitor of class I histone deacetylases (HDAC)^38^. Since HDAC inhibitors can block cardiac fibrosis and thus improve diastolic function^39,40^, elevated 3-HB serum levels might be beneficial for the failing heart. Along these lines, it would be important to examine whether ketogenic diet-induced ketosis, reported to exert positive effects on cardiovascular risk in some but not all studies^41^, would be beneficial or detrimental in AHF.

Increased serum citrate levels in patients who died within three months after onset of AHF further substantiate an increased supply of the liver and other tissues with fatty acids. Under this condition, an augmented fatty acid ß-oxidation gives rise to the increased levels of TCA cycle intermediates, including citrate. Citrate is a known potent allosteric inhibitor of pyruvate dehydrogenase which is why increased intracellular citrate levels redirect the conversion of pyruvate from acetyl coenzyme A to lactate, consequently leading to increased lactate plasma levels^42,43^. Indeed, the lactate serum levels were higher in AHF patients who died within three months after onset of AHF compared to those who survived and were significantly positively correlated with citrate serum levels (r = 0.37, p < 0.001). Besides high intracellular citrate levels, diminished oxygen supply to the tissue due to a decreased tissue perfusion and congestion, which are both hemodynamic hallmarks of HF, augments anaerobic glycolysis and lactate production^44^. In the present study, the association of lactate serum levels with 3-month mortality did not remain significant (p = 0.061) after adjusting for parameters which are associated with mortality in HF patients. This is in contrast with findings of a recent study that showed a significant association of lactate with 1-year mortality in AHF patients^45^. A possible explanation for this discrepancy is that lactate may have more of a long-term rather than a short-term prognostic capacity for mortality in AHF.

The serum levels of tyrosine at admission were also higher in patients who died within three months compared to those who were alive three months after onset of AHF. In the present study, tyrosine serum levels were significantly positively correlated with the serum levels of NT-proBNP, IL-6, and 2-HB (r = 0.24, P = 0.007), the markers of HF severity, inflammation, and oxidative stress, respectively, which are known to be associated with increased muscle proteolysis and protein turnover^46,47^. However, the fact that the serum levels of other amino acids were similar in the two patient groups presents an argument against the breakdown of proteins in the skeletal muscle being responsible for increased tyrosine serum levels in AHF patients that did not survive for three months. Therefore, the decreased uptake and use of tyrosine as a substrate for the biosynthesis of various biological molecules, including thyroid hormones, catecholamines, neurotransmitters, or serum proteins^48^, may better explain higher tyrosine serum levels in the group of patients who died within three months after onset of AHF. We observed a significant positive correlation of the tyrosine serum levels with AST and a significant negative correlation with serum fibrinogen levels in the present study. These strongly argue for a contribution of an impaired liver function and hepatocyte damage, possibly due to hypoperfusion and/or congestion, to the increased tyrosine serum levels. This is in line with results of previous studies showing that a decreased liver function and/or damage to the liver result in increased serum levels of tyrosine and other aromatic amino acids^49^.

Besides tyrosine, we observed positive correlations of 3-HB, 2-HB, lactate, and citrate with serum levels of liver transaminases, which are known to be increased as a consequence of hepatocyte damage due to reduced perfusion^14^. This suggests that not only the increased production but also a lower uptake by the liver as well as an augmented release from damaged hepatocytes likely contribute to the increased serum levels of those metabolites^44^.

The serum levels of citrate and 3-HB were negatively correlated with GFR, suggesting that the renal excretion might be factor in the regulation of the serum levels of these metabolites in AHF. Alternatively, the negative relationship between the levels of these metabolites and GFR may conceivably be a consequence of their opposite regulation by the HF pathophysiology, which most likely also explains the negative correlations of both citrate and tyrosine with various serum lipids, BMI, and MAP, which are frequently decreased in HF^50^.

This study is not free of limitations: Due to the study design we could not examine the mechanistic relationship between the underlying pathophysiological processes and the serum levels of the identified metabolites. In addition, our metabolic profiles are just a snap shot of the patients’ metabolic state at hospital admission without capturing dynamic changes in the levels of the metabolites during hospitalization. Thus we are unable to assess any temporal development or the impact of therapeutic interventions. Furthermore, we have no data on the patients’ nutritional state, i.e. whether and how long they were fasting before blood collection. Moreover, we profiled only water-soluble metabolites without addressing lipid metabolites. Finally, because the statistical power of our analyses is affected by the moderate number of available serum samples (n = 130), our results need to be confirmed in further and larger studies.

We conclude that serum levels of 2-HB, 3-HB, tyrosine, and citrate are associated with increased 3-month mortality in AHF patients and might thus be of prognostic value in AHF.

## Methods

### Study design and patients

Details of the study and its patient cohort have already been described in depth^21–24^. We conducted a prospective observational single-center study including consecutive hospitalized AHF patients. Written informed consent was obtained from all patients. The study was conducted in adherence to the ethical guidelines of the Declaration of Helsinki^51^, and was approved by the Ethics Committees of the University Hospital Centre Sisters of Charity, Zagreb, Croatia and the Medical University of Graz, Austria. The patients were treated by local standard operating procedures outlined by the ESC Guidelines for AHF^3,52^.

### Laboratory procedures

The blood sampling and laboratory methods have already been described in previous reports on our AHF cohort^21–24^.

### NMR metabolic profiling

To remove serum proteins and to quench enzymatic reactions in the samples 200 µL serum were mixed with 400 µL methanol and stored at −20 °C for 7 days until further processing. Afterwards the samples were spun at 17949 rcf at 4 °C for 30 minutes. Supernatants were lyophilized and mixed with 500 μL of NMR buffer in D_2_O and transferred to 5 mm NMR tubes. Metabolites were measured as described previously^53^.

Spectral acquisition was performed on a 600 MHz Bruker Avance Neo NMR spectrometer equipped with a TXI 600S3 probehead and processing was performed as previously described^53^. Spectra pre-processing and data analysis have been carried out as previously described^54^ using Principle Component Analysis (PCA), O-PLS-DA^55^, and all associated data consistency checks as well as 7-fold cross-validation. In order to validate the statistical significance of the determined differences between patients that did and did not survive three months, the quality assessment statistic Q^2^ is reported. This measure provides information about cross-validation and is a qualitative measure of consistency between the predicted and original data, with a maximum value of 1. Processed spectra were imported into MestreNova 12.0.2 in order to quantify metabolites of interest. Glucose quantification was performed using Chenomx Professional 8.0 with the existing Chenomx library.

### Statistical analyses

Patients’ baseline characteristics, laboratory parameters, and metabolite serum levels were descriptively analyzed using absolute and relative frequencies or median and range. Differences between patients who did and did not survived the first three months after onset of AHF were assessed either by Fisher’s exact or the Mann-Whitney U test. Correlations between the metabolites and various clinical and laboratory parameters were determined by the Spearman correlation coefficient. Metabolite concentrations were used to perform an ROC curves analysis in MetaboAnalyst 4.0^56^. Additionally, the impact of the metabolites on 3-month mortality was examined using univariable as well as multivariable logistic regression analyses. The latter was adjusted for age, sex, BMI, T2DM, NT-proBNP, GFR, MAP as well as LDL cholesterol. Odds ratios (OR) along with the respective 95% confidence intervals (CI) are presented. In order for the resulting ORs to be on an interpretable scale, all metabolites were log-transformed for the regression analyses. The variance inflation factor was used to assess the degree of multi-collinearity in the models. R version 3.4.4 was used for the statistical analyses.

All methods were carried out in accordance with the relevant guidelines and regulations.

## Supplementary information

Supplementary Table 1

## Data Availability

All data generated or analysed during this study are included in this manuscript.
