# Supplementary material for: Serum Concentrations of Citrate, Tyrosine, 2- and 3- Hydroxybutyrate are Associated with Increased 3-Month Mortality in Acute Heart Failure Patients
Source: Sci Rep. 2019 May 1;9:6743. doi: 10.1038/s41598-019-42937-w (PMC6494857; doi:10.1038/s41598-019-42937-w)
Supplement: Supplementary file 1 — Supplementary Table 1 [file 41598_2019_42937_MOESM1_ESM.pdf]

Serum Concentrations of Citrate, Tyrosine, 2- and 3- Hydroxybutyrate are Associated with Increased 3-Month Mortality in Acute Heart Failure Patients

Sarah Stryeck, Michaela Gastrager, Vesna Degoricija, Matias Trbušić, Ines Potočnjak, Bojana Radulović, Gudrun Pregartner, Andrea Berghold, Tobias Madl and Saša Frank

Supplementary Table 1. Comorbidities of AHF patients who died compared to those who survived the first three months after onset of AHF

	All (N=130)	Alive (N=98)	Dead (N=32)	p-value
**Hyperlipidemia**	52 (40%)	39 (39.8%)	13 (40.6%)	1.000
**Hypercholesterolemia**	51 (39.2%)	39 (39.8%)	12 (37.5%)	1.000
**Hypertension**	117 (90.0%)	90 (91.8%)	27 (84.4%)	0.306
**T2DM**	68 (52.7%)	53 (54.6%)	15 (46.9%)	0.541
**COPD**	34 (26.2%)	24 (24.5%)	10 (31.2%)	0.490
**CKD**	41 (31.5%)	27 (27.6%)	14 (43.8%)	0.124
**CM**	95 (73.1%)	68 (69.4%)	27 (84.4%)	0.113
**ACS**	17 (13.1%)	14 (14.3%)	3 (9.4%)	0.562

Data are presented as n (%). Differences between AHF patients who died and those who survived the first three months after onset of AHF were tested with Fisher's exact test.
ACS, acute coronary syndrome; AHF, acute heart failure; CKD, chronic kidney disease; CM, cardiomyopathy; COPD, chronic obstructive pulmonary disease; T2DM, Type 2 Diabetes Mellitus;
